# Clinical Audit of Venous Thromboembolism (VTE) Risk Assessment and Guideline Compliance in Surgical Practice

**DOI:** 10.7759/cureus.91288

**Published:** 2025-08-30

**Authors:** Ali Bashir Mohammed Ahmed, Awad Abdalla Widaa Mohamed, Mustafa Mohamed, Elmonzir Hamed Mohammed Elghorashi, Noha Abdelhalim Siddig Rikabi, Mukhtar Magdi Mukhtar Bastawi, Abdelrahman Edris Osman Ali, Abdulwahab Mustafa, Asim Faisal Awad Ibrahim, Islam Hamza Haroun Ahmedelamin, Alnazeer Y Abdelbagi, Mohamed Yasir Fateh Alrhman, Mutwalli Mohamed Awadallah Yousif, Alkhansaa Mohamed Ali Mohamed, Hussam Mohamed Ahmed Elawad, Walaa Osman Abdelhameed Mohamed, Mohammed Yousif Khalid Makki, Hibatallah Ahmed, Mohmed H Ahmed Mohmed, Mubarak Mohamed Ali

**Affiliations:** 1 General Surgery, Prince Othman Digna Teaching Hospital, Port Sudan, SDN; 2 General Surgery, Port Sudan Teaching Hospital, Port Sudan, SDN; 3 Internal Medicine, Prince Othman Digna Teaching Hospital, Port Sudan, SDN; 4 General Surgery, Hayat National Hospital, Unaizah, SAU; 5 Medicine and Surgery, Sudan International University, Khartoum, SDN; 6 General Surgery, Port Sudan Doctors Hospital, Port Sudan, SDN; 7 Anatomy, University of Khartoum, Khartoum, SDN; 8 Medicine, National Ribat University, Khartoum, SDN; 9 Emergency Medicine and Surgery, King Fahad Hospital, Al Bahah, SAU; 10 Emergency Medicine, Kassala Teaching Hospital, Kassala, SDN; 11 Emergency Medicine, Omdurman Military Hospital, Khartoum, SDN; 12 Internal Medicine, International University of Africa, Khartoum, SDN; 13 Medicine, University of Gezira, Wad Madani, SDN; 14 General Surgery, University of Gezira, Wad Madani, SDN

**Keywords:** caprini score, patient education, port sudan doctors hospital, prophylaxis, quality improvement

## Abstract

Background

Venous thromboembolism (VTE) remains a major cause of preventable morbidity and mortality among surgical inpatients. Despite established international guidelines, adherence to prophylaxis protocols is often suboptimal in clinical practice.

Objective

To evaluate and improve adherence to VTE risk assessment and prophylaxis guidelines among surgical patients at Port Sudan Doctors Hospital through a structured clinical audit.

Methods

A two-cycle prospective clinical audit was conducted. The first cycle (September 2024) involved a retrospective review of 50 surgical inpatient records and a gap analysis through staff interviews and observations. A two-month intervention followed, introducing a standardized VTE risk assessment tool, staff training, and integration of clinical workflows. The second cycle (January-April 2025) included a prospective review of 41 patient records and qualitative staff feedback. Data were analyzed descriptively and thematically.

Results

Compliance with VTE prophylaxis guidelines improved significantly from 6% in the first cycle to 85.4% in the second. Patient education increased from 4% to 95.1%, and proper documentation of prophylaxis duration improved from 6% to 95.1%. The use of structured risk assessment tools (e.g., Caprini score) increased from 7.1% to 50%, while inappropriate pharmacological prophylaxis for low-risk patients was eliminated. Staff engagement and usability of tools were also enhanced.

Conclusion

Targeted interventions, including standardized risk assessment and focused staff education, significantly improved VTE prophylaxis practices. Sustained improvements will require continued auditing, digital integration of tools, and institutional support to promote long-term adherence and patient safety.

## Introduction

Venous thromboembolism (VTE), encompassing both deep vein thrombosis (DVT) and pulmonary embolism (PE), is among the most preventable causes of serious complications and in-hospital mortality worldwide [1]. Surgical patients, in particular, face heightened vulnerability due to a convergence of factors including immobility, surgical trauma, and pro-inflammatory and pro-coagulant responses, all of which exacerbate the classical Virchow’s triad of venous stasis, vascular injury, and hypercoagulability [2].

The consequences of VTE extend beyond immediate clinical events, exerting a considerable toll on healthcare systems globally. Many affected individuals suffer from persistent sequelae such as chronic thromboembolic pulmonary hypertension, post-thrombotic syndrome, and ongoing functional impairments, all of which contribute to long-term health deterioration and escalating healthcare demands [3,4].

Extensive evidence from randomized controlled trials and systematic reviews confirms that appropriate pharmacological prophylaxis can reduce the risk of VTE by up to 70% in hospitalized populations [5]. Leading international bodies, including the American College of Chest Physicians (ACCP), the National Institute for Health and Care Excellence (NICE), and the American Society of Hematology (ASH), have developed comprehensive guidelines to guide prevention efforts [6-8].

Despite the clarity of these protocols, real-world adherence remains suboptimal. Studies in healthcare improvement emphasize that passive dissemination of guidelines alone is insufficient. Instead, strategies that integrate clinician education with real-time audit-feedback mechanisms and clinical decision support tools show markedly superior results. Evidence suggests that when such comprehensive interventions are implemented, adherence rates can reach 90-95%, whereas passive strategies often result in compliance rates below 50% [1].

Yet, consistent evaluation and implementation of both pharmacological and mechanical VTE prophylaxis remain fragmented across many institutions. Mechanical prophylactic measures, in particular, are often inconsistently applied. Against this backdrop, this clinical audit at Port Sudan Doctors Hospital was designed to critically assess how thoroughly VTE risk assessments are being conducted for surgical inpatients, evaluate the appropriateness of prophylactic measures provided, and ultimately promote greater conformity with established best practices to reduce preventable thromboembolic complications.

## Materials and methods

Study design and setting

This prospective clinical audit was conducted at Port Sudan Doctors Hospital, Port Sudan, Sudan, a regional referral center serving a diverse population with a high surgical volume. The audit was designed to evaluate and enhance adherence to VTE prophylaxis guidelines among surgical inpatients. The focus was on the adequacy of risk assessment and the appropriateness of prophylactic measures as part of perioperative care.

Study phases and interventions

First Cycle (Baseline Assessment and Gap Analysis)

The initial phase started in September 2024, involved a systematic review of current practices related to VTE prevention among surgical patients. Through retrospective examination of inpatient medical records, multiple deficiencies were identified, including the inconsistent application of VTE risk assessment tools and inadequate documentation or administration of prophylactic measures. To gain deeper insight into these issues, a gap analysis was conducted. This process included structured interviews and informal discussions with surgeons, junior doctors, nurses, and clinical pharmacists to identify workflow barriers, knowledge gaps, and systemic challenges. Observational rounds were also carried out to directly assess bedside practices and policy implementation. The findings underscored the need for standardized VTE risk assessment protocols, consistent documentation practices, and enhanced clinical education to improve awareness and adherence to VTE prevention measures.

Intervention

Following the baseline analysis, a two-month multifaceted intervention was developed and implemented. Central to this strategy was the introduction of a standardized VTE risk assessment form based on international guidelines, adapted to suit the hospital’s clinical workflow. The form was incorporated into the surgical admission protocol to ensure timely and systematic risk stratification.

To support uptake, a series of structured training sessions was organized for clinical staff, focusing on the pathophysiology of VTE, the rationale for prophylaxis, guideline recommendations according to the ACCP and the NICE [6,7], and the correct use of the risk assessment tool. These educational sessions employed a blended learning approach, combining lectures, interactive case-based discussions, and bedside simulations. Supplementary materials, including pocket reference cards, visual posters, and a digital quick-check tool, were disseminated across wards to reinforce key messages.

To further promote compliance, the VTE protocol was integrated into daily ward rounds, and senior nurses, along with surgical residents, were designated as protocol champions to monitor adherence and provide real-time support. Regular feedback loops and peer review discussions were encouraged to maintain momentum and foster a culture of accountability.

Second Cycle (Post-intervention Review and Impact Evaluation)

Two months following the intervention, a second cycle of data collection was initiated to evaluate its effectiveness between January and April 2025. A total of 41 surgical patient records were randomly selected and reviewed using the newly implemented assessment framework. The review focused on documentation completeness, the accuracy of risk stratification, and the suitability of prescribed prophylactic measures. In addition, informal interviews were conducted with healthcare staff to gather qualitative feedback on the practicality, clarity, and integration of the new system into routine practice.

This phase also allowed for refinement of the risk assessment tool based on end-user feedback, leading to minor modifications aimed at improving usability and alignment with workflow patterns. Adjustments included clearer formatting of contraindication sections and integration with electronic documentation for easier access and compliance tracking.

Data analysis

Collected data were evaluated by three independent reviewers, two surgical residents, and one internal medicine physician who were trained on a uniform evaluation protocol to minimize inter-rater variability. Each record was assessed for adherence to VTE risk assessment and prophylaxis documentation standards. Descriptive statistics were used to compare pre- and post-intervention compliance rates, while thematic analysis of staff feedback was conducted to identify barriers and enablers to implementation.

While the use of trained reviewers and structured tools strengthened methodological rigor, the absence of formal blinding during record evaluation represents a limitation that could introduce bias. Future studies may benefit from incorporating blinded assessments and automated data capture to enhance objectivity and scalability.

Evaluation of doctors’ practices

In parallel with the review of patient records, the audit incorporated a structured evaluation of doctors’ clinical practices concerning VTE risk assessment and prophylaxis. A total of 14 doctors were assessed during the first audit cycle, and 12 doctors in the second cycle. All participants were directly involved in the perioperative care of surgical inpatients.

Each doctor completed a structured, self-administered questionnaire that examined key aspects of their clinical approach, including the method used for VTE risk assessment (e.g., clinical judgment vs. validated scoring tools), choice of prophylactic strategy for different risk categories, duration of prescribed prophylaxis, and frequency of patient education on VTE prevention. The questionnaires were collected anonymously to minimize reporting bias and encourage candid responses. The data were subsequently analyzed to compare practice patterns before and after the intervention, providing insights into the behavioral impact of the implemented changes.

Evaluation

The evaluation framework focused on assessing intervention fidelity, implementation outcomes, and clinical relevance. This included measuring staff engagement, changes in practice patterns, and perceived usefulness of the tools introduced. The audit cycle was designed not only to improve clinical practice but also to inform broader quality improvement efforts related to patient safety and thromboprophylaxis.

Ethical considerations

This study received ethical approval from the Institutional Audit Committee at Port Sudan Doctors Hospital (Approval Code: 2024/CA0178). All data collected were anonymized prior to analysis to protect patient confidentiality. While formal informed consent was not required due to the audit nature of the project, all staff participants were informed of the purpose and scope of the intervention. Future iterations of this audit may consider more explicit documentation of verbal consent and expanded stakeholder engagement to strengthen ethical transparency and accountability.

## Results

Comparison of audit findings between cycles 1 and 2

This result compares the key findings from the first and second cycles of the clinical audit on VTE risk assessment and guideline adherence for surgical patients, highlighting significant improvements in clinical practice and documentation (Table 1).

**Table 1:** Comparative Analysis of VTE Prophylaxis Parameters Between Two Audit Cycles at Port Sudan Doctors Hospital This table summarizes key improvements observed between the first and second audit cycles on venous thromboembolism (VTE) risk assessment and prophylaxis among surgical inpatients at Port Sudan Doctors Hospital. The data illustrate changes in clinical practice, patient education, documentation accuracy, and adherence to established guidelines.

Parameter		1st Cycle Audit (N = 50), N (%)	2nd Cycle Audit (N = 41), N (%)	Comments on Comparison
Guideline adherence		3 (6)	35 (85.4)	A substantial improvement in adherence to VTE prophylaxis guidelines.
Patient education		2 (4)	39 (95.1)	A dramatic increase, indicating new emphasis on patient involvement and awareness.
Average Caprini score		5.7	2.71	A lower average score may indicate a shift in patient case mix or broader inclusion.
Prophylaxis plan duration	Till discharge	2 (4)	33 (80.5)	Marked improvement in specificity; "Unspecified" duration significantly reduced.
	7-10 days	1 (2)	4 (9.8)	
	30 days	0 (0)	2 (4.9)	
	Unspecified	47 (94)	2 (4.9)	
Missing data completeness (patient ID)		12 (24)	2 (4.9)	Significant enhancement in record-keeping, especially regarding patient identifiers.

Key Improvement: Enhanced Guideline Adherence

The most significant achievement is the dramatic increase in guideline coherence, which rose from a mere 6% to 85.4%. This demonstrates a major shift towards evidence-based practice.

Prioritization of Patient Education

Patient education on VTE risk has become a standard of care, with the rate of educated patients soaring from 4% to 95.1%. This empowerment of patients is a cornerstone of modern, patient-centered healthcare.

Improved Documentation

The second cycle shows a drastic reduction in ambiguity regarding the duration of VTE prophylaxis. The proportion of plans with an "unspecified" duration fell from 94% to just 4.9%.

Better Data Quality

The significant decrease in missing patient IDs from 24% to 4.9% indicates an improvement in the overall quality of data collection.

Doctor response analysis

Table 2 provides a detailed, parameter-by-parameter comparison of doctors’ responses collected during cycles 1 and 2 of the clinical audit. It highlights key changes in clinical decision-making, risk assessment approaches, prophylaxis planning, and patient education practices following the implementation of targeted interventions.

**Table 2:** Comparative Analysis of Doctors’ Practices on VTE Risk Assessment and Prophylaxis Across Two Audit Cycles at Port Sudan Doctors Hospital This table presents a comparison of responses from doctors involved in venous thromboembolism (VTE) prevention during two audit cycles at Port Sudan Doctors Hospital. It highlights changes in professional roles, risk assessment methods, prophylaxis strategies, and patient education practices following targeted interventions. SC: subcutaneous

Parameter		Cycle 1 Findings (n=14), n (%)	Cycle 2 Findings (n=12), n (%)	Comment Improvement
Level of practice	Medical officer	11 (78.6)	7 (58.3)	The proportion of registrars participating increased in the second cycle. This change in the respondent mix is an important context for the results.
	Registrar	3 (21.4)	5 (41.7)	
Primary method of VTE risk assessment	Clinical judgement	10 (71.4)	7 (58.3)	Significant improvement: There was a 36.9 percentage point increase in the use of structured guidelines, showing a shift to more reliable, evidence-based methods.
	Structured guidelines	3 (21.4)	5 (41.7)	
Method for risk stratification (high vs. low)	Caprini score	1 (7.1)	6 (50)	Excellent improvement: The adoption of the specific, validated Caprini score increased from 7.1% to 50%, standardizing how risk is calculated.
	Left blank	6 (42.9)	3 (25)	
Use of pharmacological prophylaxis (e.g., SC heparin or rivaroxaban) in patients classified as low risk.		6 (42.9)	0 (0)	Significant improvement: There was a complete (100%) elimination of inappropriate pharmacological prophylaxis for low-risk patients, aligning practice with safety guidelines.
Extended pharmacological prophylaxis (e.g., SC heparin for 30 days) in high-risk patients.		3 (21.4)	8 (66.7)	Good improvement: Adherence to guideline-recommended extended (30-day) prophylaxis for high-risk patients more than tripled, rising from 21.4% to 66.7%.
Patient education provided	Always	7 (50)	9 (75)	Significant improvement: The rate of doctors who always educate their patients increased by 25 percentage points, marking a positive shift towards better patient communication.
	Sometimes	7 (50)	3 (25)	

Key Achievements in Numbers

Assessment standardization: The use of structured guidelines rose from 21.4% to 58.3%. The specific use of the Caprini score for risk stratification surged from 7.1% to 50%.

Improved safety and appropriateness: Inappropriate prescription of anticoagulants for low-risk patients was reduced from 42.9% to 0%. For high-risk patients, adherence to extended 30-day prophylaxis increased from 21.4% to 66.7%.

Better patient engagement: The proportion of doctors consistently educating their patients about VTE risk increased from 50% to 75%.

## Discussion

This clinical audit evaluated the effectiveness of targeted quality improvement interventions in enhancing adherence to VTE prophylaxis guidelines among surgical patients at Port Sudan Doctors Hospital. The data from cycles 1 and 2 revealed substantial improvements in both clinical practice and documentation.

Notably, adherence to VTE prophylaxis guidelines increased from 6% to 85.4%, a finding consistent with previous research demonstrating that structured interventions such as staff education, audits, and the introduction of standardized protocols can significantly improve guideline compliance in surgical settings [9]. Hospital-acquired VTE remains one of the most preventable causes of morbidity and mortality among inpatients, and systematic risk assessment is considered a cornerstone of prophylaxis programs [10].

An equally encouraging development was the rise in patient education, which soared from 4% in cycle 1 to 95.1% in cycle 2, reflecting a strengthened emphasis on patient-centered care. Literature has shown that patients who are educated about their VTE risk and the rationale for prophylaxis are more likely to adhere to therapy and recognize early symptoms of thromboembolic events, ultimately improving safety and outcomes [11,12]. In addition to better patient involvement, there was a marked shift among physicians toward structured risk assessment.

The use of the Caprini score increased from 7.1% to 50%, while reliance solely on clinical judgment declined. This mirrors findings from global studies showing that the implementation of validated tools like the Caprini model enhances appropriate prophylaxis and reduces variability in risk classification [11].

Furthermore, documentation improved considerably. In cycle 1, 94% of prophylaxis plans lacked a clearly stated duration, compared to just 4.9% in cycle 2. Proper documentation of prophylaxis duration is essential to ensuring appropriate therapy length, and its improvement reflects enhanced clinical vigilance and adherence to best practices [10]. The audit also revealed a complete elimination of inappropriate pharmacologic prophylaxis for low-risk patients, dropping from 42.9% to 0% and a significant rise in extended 30-day prophylaxis for high-risk patients, from 21.4% to 66.7%. These trends reflect improved application of risk-based decision-making, in line with evidence-based guidelines [13].

However, despite this progress, 41.7% of clinicians in the second cycle continued to rely on clinical judgment rather than structured tools. This indicates that further reinforcement, such as embedding the Caprini score into mandatory preoperative checklists or digital workflows, may help standardize practice and close the remaining compliance gap [14]. Additionally, the reduction in the average Caprini score from 5.7 to 2.71 may suggest a change in the patient profile in the second cycle, possibly reflecting the inclusion of more low-risk surgical patients. Stratified analysis of high-risk subgroups could help ensure that improvements are consistent across all risk categories.

Beyond these measured improvements, the audit also highlights the vital role of multidisciplinary engagement in driving clinical quality improvement. The involvement of surgeons, nurses, and administrative staff fostered a shared culture of accountability and continuous learning. This collective commitment is crucial for sustaining improvements beyond the audit period.

Improved documentation and patient education practices demonstrate enhanced communication both within clinical teams and with patients, promoting safer care and patient empowerment. Despite resource limitations, these gains demonstrate that simple, targeted interventions can lead to meaningful changes in clinical behavior and patient outcomes.

Nevertheless, sustaining these gains will require integrating risk assessment tools into routine workflows and institutional policies, alongside ongoing training and audit cycles to maintain momentum. Additionally, the observed decrease in average Caprini scores across cycles suggests the need for future audits to analyze risk groups separately, ensuring high-risk patients consistently receive appropriate prophylaxis.

Finally, the relatively small sample size limited the ability to formally correlate improvements in clinical practice with practitioner education levels, which would have added valuable insight into the mechanisms of change. Future studies should include knowledge assessments alongside audits to better understand how education influences clinical behavior.

It is important to note that this clinical audit has a number of limitations. The results may not apply to other institutions or patient populations due to the limited sample size, which is the primary limitation. Also, the audit didn't check if there was a statistical correlation between the healthcare practitioners' knowledge or education levels and the improvements in patient education, documentation practices, and adherence to guidelines between the two cycles. More thorough study and greater proof of causation could have resulted from establishing such a correlation. But this kind of inferential analysis couldn't be done with such a small sample size. Additionally, documentation was crucial to this audit, and documentation may not necessarily represent real clinical practice. In conclusion, it is impossible to determine if the changes made are sustainable due to the absence of a long-term follow-up.

## Conclusions

This audit demonstrated that targeted interventions such as standardized risk assessment, staff education, and improved documentation significantly enhanced VTE prophylaxis practices among surgical inpatients. Despite a limited sample size, improvements in guideline adherence and patient education were notable. Sustaining these gains will require ongoing audits, integration of tools like the Caprini score into routine practice, and institutional support. Future audits should include knowledge assessments to better understand how practitioner education impacts clinical behaviour.
